# Role of Oxidative Stress in the Pathophysiology of Pneumococcal Meningitis

**DOI:** 10.1155/2013/371465

**Published:** 2013-05-09

**Authors:** Tatiana Barichello, Jaqueline S. Generoso, Lutiana R. Simões, Samuel G. Elias, João Quevedo

**Affiliations:** ^1^Laboratório de Microbiologia Experimental, Programa de Pós-Graduação em Ciências da Saúde, Unidade Acadêmica de Ciências da Saúde, Universidade do Extremo Sul Catarinense, 888806-000 Criciúma, SC, Brazil; ^2^Instituto Nacional de Ciência e Tecnologia Translacional em Medicina (INCT-TM), Programa de Pós-Graduação em Ciências da Saúde, Unidade Acadêmica de Ciências da Saúde, Universidade do Extremo Sul Catarinense, 88806-000 Criciúma, SC, Brazil; ^3^Núcleo de Excelência em Neurociências Aplicadas de Santa Catarina (NENASC), Programa de Pós-Graduação em Ciências da Saúde, Unidade Acadêmica de Ciências da Saúde, Universidade do Extremo Sul Catarinense, 88806-000 Criciúma, SC, Brazil; ^4^Laboratório de Neurociências, Programa de Pós-Graduação em Ciências da Saúde, Unidade Acadêmica de Ciências da Saúde, Universidade do Extremo Sul Catarinense, 888806-000 Criciúma, SC, Brazil

## Abstract

Pneumococcal meningitis is a life-threatening disease characterized by an acute purulent infection affecting the pia mater, the arachnoid, and the subarachnoid spaces. *Streptococcus pneumoniae* crosses the blood-brain barrier (BBB) by both transcellular traversal and disruption of the intraepithelial tight junctions to allow intercellular traversal. During multiplication, pneumococci release their bacterial products, which are highly immunogenic and may lead to an increased inflammatory response in the host. Thus, these compounds are recognized by antigen-presenting cells through the binding of toll-like receptors. These receptors induce the activation of myeloid differentiation factor 88 (MyD88), which interacts with various protein kinases, including IL-1 receptor-associated kinase-4 (IRAK4), which is phosphorylated and dissociated from MyD88. These products also interact with tumor necrosis factor receptor-associated factor 6 dependent signaling pathway (TRAF6). This cascade provides a link to NF-**κ**B-inducing kinase, resulting in the nuclear translocation of NF-**κ**B leading to the production of cytokines, chemokines, and other proinflammatory molecules in response to bacterial stimuli. Consequently, polymorphonuclear cells are attracted from the bloodstream and then activated, releasing large amounts of NO^•^, O_2_^*•*^, and H_2_O_2_. This formation generates oxidative and nitrosative stress, subsequently, lipid peroxidation, mitochondrial damage, and BBB breakdown, which contributes to cell injury during pneumococcal meningitis.

## 1. Introduction

Pneumococcal meningitis is the most complex and serious infection of the central nervous system (CNS) that is associated with neurological sequelae [1]. The host immune response, through the production of cytokines and chemokines and the migration of leukocytes, is the first line of defense in response to bacterial infection [2]. In addition, polymorphonuclear leukocytes produce nitric oxide (NO^•^), superoxide anion radicals (O_2_^−•^), and hydrogen peroxide (H_2_O_2_). O_2_^−•^ and NO^•^ can lead to the formation of peroxynitrite (ONOO), which is a strong oxidant [3]. This oxidant exerts cytotoxic effects on endothelial cells [4], increases the permeability of the BBB, induces the peroxidation of lipids, and induces many other complex interactions that seem to be involved in the pathophysiology of pneumococcal meningitis [3].

The aim of this review is to summarize the current knowledge of the relevant pathophysiological steps of pneumococcal meningitis: (a) the crossing of the pneumococcus through the BBB; (b) the activation of innate immune system mechanisms; (c) the migration of leukocytes and (d) the induction of oxidative and nitrosative stress in the context of pneumococcal meningitis.

## 2. Microbial Traversal of the Blood-Brain Barrier

The CNS is protected by a bony skull, the leptomeninges, the blood-brain barrier (BBB), and the blood-cerebrospinal fluid barrier [5]. The BBB is formed by microvascular endothelial cells, astrocytes, and pericytes. This barrier acts by controlling the exchange of substances into and out of the brain [6] and thereby protects the brain from toxins and pathogens [5]. *S. pneumoniae* crosses the BBB through both transcellular traversal and paracellular traversal [6, 7]. In transcellular traversal, the pathogen interacts with cell-wall phosphorylcholine and the platelet-activating-factor (PAF) receptor. In addition, the protein C (PspC) pneumococcal surface binds to both the laminin receptor and the polymeric Ig receptor (pIgR), which are located on brain microvascular endothelial cells [8]. Later, the pathogen transmigrates through endothelial cells to the basolateral side without any evidence of disruption of intercellular tight junctions [5, 6]. 

**Figure 1:**
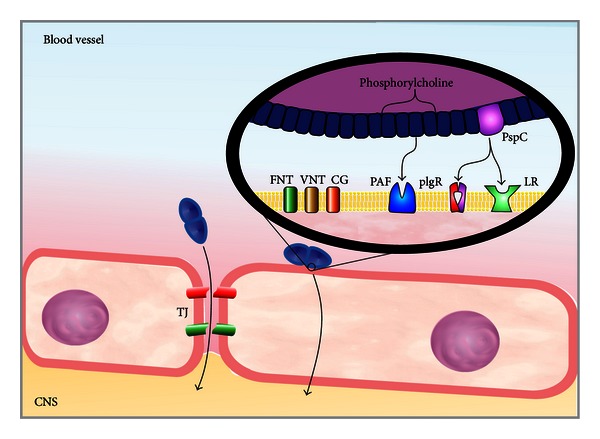
Mechanisms of microbial traversal of the BBB. *S. pneumoniae* crosses the BBB through transcellular traversal and paracellular traversal.

Paracellular traversal involves the penetration of bacteria between barrier cells with or without evidence of tight-junction disruption [6]. Both the host immune response and bacterial virulence factors, such as pneumolysin, and the ability of pneumococci to bind to fibronectin [9], vitronectin, and collagen in the extracellular matrix, act together to increase the permeability of the BBB [10, 11]. This interaction facilitates the passage of the microorganism into the brain [1] Figure 1.

## 3. Innate Immune Mechanisms of the Pneumococcal Meningitis

**Figure 2:**
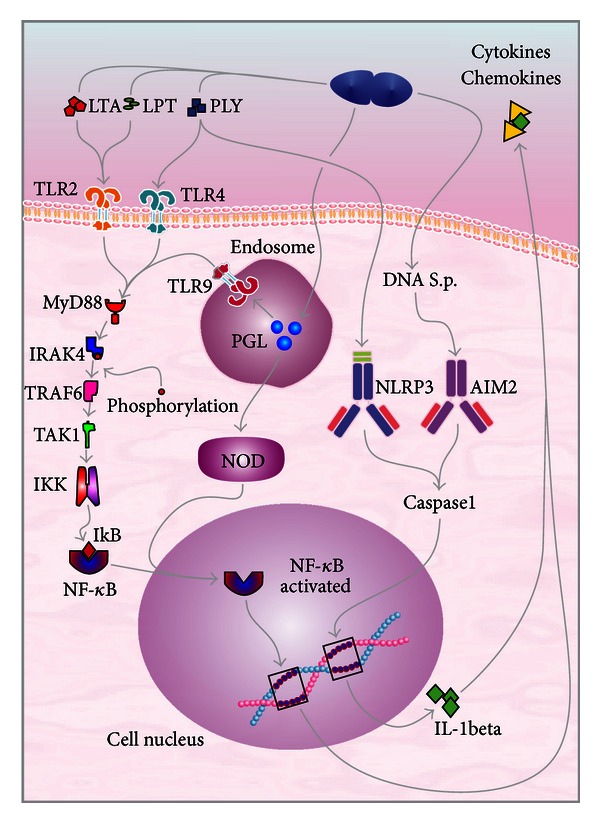
Innate immune system in pneumococcal meningitis infection. The majority of TLRs utilize a common intracellular adapter protein known as myeloid differentiation factor 88 (MyD88): it activates IRAK, which is phosphorylated and dissociated from MyD88. Thus, it interacts with the tumor necrosis factor receptor-associated factor 6 dependent signaling pathway (TRAF6). TRAF6 stimulates to the transforming growth factor *β*-activated kinase (TAK1). TAK1 activates the IKK (Inhibitor of I*κ*B kinase), resulting in the destruction of IkB and subsequently, NF-*κ*B activation resulting in the nuclear translocation of NF-*κ*B. This cascade provides a link to NF-*κ*B-inducing kinase, resulting in the nuclear translocation of NF-*κ*B, which induces the production of cytokines, chemokines, and others proinflammatory molecules in response to bacterial stimuli.

After* S. pneumoniae* reaches the subarachnoid space, it multiplies rapidly and releases compounds, such as cell wall fragments, lipoteichoic acid, teichoic acid, pneumolysin, and peptidoglycan [1]. These compounds are highly immunogenic and may elicit an inflammatory response in the host. These immunogenic molecular determinants are better known as pathogen-associated molecular patterns (PAMPs) [12, 13]. These PAMPs are recognized by different sensors of the innate immune system called pattern recognition receptors (PRRs) [14]. These PRRs comprise toll-like receptors (TLRs), NOD-like receptors (NLRs), and DNA sensors [14, 15]. At present, there are 13 members of the TLR family described in humans and 10 described in mice. These members are separated into two broad categories. One category is expressed at the cell surface for extracellular ligand recognition. The other category is localized in the endosomal compartment for the recognition of pathogen nucleic acids [16]. Microglia express all TLRs identified to date, whereas astrocytes only express TLR1, TLR2, TLR3 and TLR9. Neurons only express TLR3, TLR7, TLR8, and TLR9, and oligodendrocytes only express TLR2 and TLR3 [15, 17]. TLR2 is activated by pneumococcal cell wall compounds, lipoteichoic acid, and lipoproteins. TLR4 is activated by pneumolysin, and TLR9 is activated by pneumococcal DNA containing CpG motifs within endosomes [14, 15]. TLR2, TLR4, and TLR9 transduce their signals through a common intracellular adapter protein known as myeloid differentiation factor 88 (MyD88) [14, 18]. Of note, the deficiency of this intracellular adapter protein in children increases their susceptibility to invasive pneumococcal infections, including meningitis [19]. MyD88 interacts with a protein kinase, IL-1 receptor-associated kinase-4 (IRAK4) [1, 20]. The IRAK4 dependent, TLRs, and IL-1Rs are vital for childhood immunity to pyrogenic bacteria, which are mainly invasive pneumococcal infections [21]. After IRAK has been phosphorylated, it is dissociated from MyD88 and interacts with tumor necrosis factor receptor-associated factor 6 dependent signaling pathway (TRAF6) [22]. TRAF6 stimulates the transforming growth factor *β*-activated kinase (TAK1), which is a MAPKKK. Thus, TAK1 activates IKK (Inhibitor of IkB kinase), which results in the destruction of IkB and the subsequent activation and nuclear translocation of NF-*κ*B [23, 24]. NF-*κ*B comprises a closely related family of transcription factors, which play a key role in the expression of genes involved in the development of accessory cell and leukocyte populations, inducing the expression of many proteins implicated in inflammation and in the immune response [25]. NF-*κ*B is also a transcriptional activator of various genes implicated in neuronal pathogenesis and in the production of cytokines and chemokines [20, 26]. The nucleotide-binding-oligomerization-domains-NOD-like receptors (NLRs) are also involved in the recognition of *S. pneumoniae* by the innate immune system. The family members consist of intracellular receptors, such as inflammasome-forming proteins (NLRPs), NLRP1, NLRP3, and NLRP6, which mediate the assembly of inflammasome complexes leading to the activation of procaspase-1. The second group of NLRs includes intracellular recognition receptors, such as NOD1/CARD4 and NOD2/CARD15. These receptors mediate the assembly of complexes that activate MAPK and NF-*κ*B signaling pathways, and they are involved in the detection of cell wall peptidoglycan [27, 28]. NLRP3 (cryopyrin) and AIM2 (absent in melanoma 2) inflammasomes are activated by pneumolysin and bacterial DNA. These inflammasomes use an adapter molecule, known as apoptosis-associated speck-like protein (ASC), which is a key component of multimeric protein complexes that mediate inflammation and host defenses [29]. NLRP3 and AIM2 promote caspase-1 activation and the subsequent conversion of pro-IL-1*β* into mature IL-1*β* in pneumococcal meningitis [30]. Furthermore, pneumolysin activates the NLRP3 inflammasome and promotes the production of the proinflammatory cytokines independently of TLR4 [31], Figure 2.

## 4. Leukocyte Migration

**Figure 3:**
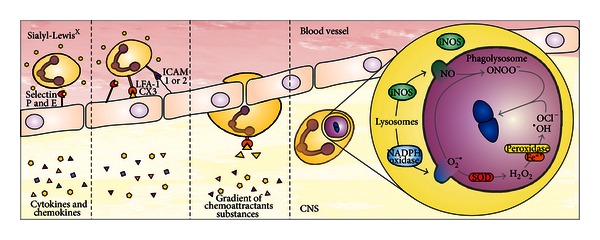
Leukocyte migration. Leukocytes leave the blood and migrate to sites of infection. Sialyl-Lewis^X^ on leukocytes binds to selectins P and E on endothelial cells. This binding becomes stronger when CXCL-8 binds to its specific receptor on neutrophils, triggering the production of integrin LFA-1 and CX3 (mac-1). Inflammatory cytokines, such as TNF-*α*, are also necessary to induce expression of the adhesion molecules ICAM-1 and ICAM-2. The interaction between endothelial cells and ICAM-1 allows the passage of neutrophils along a gradient of chemoattractants substances.

Pneumococcal compounds are proinflammatory mediators that induce an innate immune response that activates NF-*κ*B and subsequently triggers the production of proinflammatory cytokines and chemokines and the expression of co-stimulatory molecules [32]. In response, neutrophils leave the blood and migrate to sites of infection. Sialyl-Lewis^X^ on leukocytes binds to selectins P and E on endothelial cells. This binding becomes stronger when CXCL-8 binds to its specific receptor on neutrophils, which triggers the production of integrin LFA-1 and CX3 (mac-1). Inflammatory cytokines, such as TNF-*α*, are also necessary to induce expression of adhesion molecules ICAM-1 and ICAM-2. The link between endothelial cells and ICAM-1 allows the passage of neutrophils along a gradient of chemoattractants substances [33, 34], Figure 3. Consistent with the polymorphonuclear migration, as explained previously, TNF-*α* is produced mainly in the first 6 to 24 hours after pneumococcal meningitis induction [35]. Patients with bacterial meningitis also have increased the levels of TNF-*α* in the CSF early in the course of the disease [36]. In bacterial meningitis, approximately 90% of the migrating leukocytes are neutrophilic granulocytes [37]. However, blocking the accumulation of leukocytes in cerebrospinal fluid augments bacteremia and lethality in experimental pneumococcal meningitis [38].

Initially, phagocytized pathogens are internalized in the phagosome. The phagosome is acidified by fusion with lysosomes, becoming a phagolysosome. In this period, high amounts of reactive oxygen species (ROS) and reactive nitrogen species (RNS) are formed [39]. The relevant antimicrobial systems of phagocytic cells are the NADPH phagocytic oxidase and inducible nitric oxide synthase (iNOS) pathways, which are expressed in neutrophil and macrophage cells [40]. Macrophages and neutrophils produce NO^•^, O_2_^−•^, and H_2_O_2_. NO^•^ is produced by iNOS_2_, and O_2_^−•^ is produced by NADPH oxidase. O_2_^−•^ and NO^•^ can lead to the formation of ONOO^−^ [3], which is a strong oxidant that exerts cytotoxic effects on endothelial and vascular smooth muscle cells [4]. Moreover, chemical and enzymatic reactions produce a variety of toxic chemical agents; O_2_^−•^ is converted by the enzyme superoxide dismutase (SOD) into H_2_O_2_ [38, 39]. H_2_O_2_ can kill the microorganisms and also be converted by the peroxidase enzyme in the presence of Fe^2+^ into hypochlorite (OCl^−^) and hydroxyl radicals (^•^OH), which are microbicides [3, 39, 41].

## 5. Oxidative Stress in the Context of Pneumococcal Meningitis

During pneumococcal meningitis, RNS and ROS are produced by resident immune cells of the brain as part of the host response to invasive bacterial infections [1, 42]. Furthermore, ROS are produced in greater quantities in neutrophils than in macrophages; however, macrophages produce more RNS than neutrophils [43]. *S. pneumoniae* also produces H_2_O_2_, which interacts with NO^•^ forming ONOO^−^ [44, 45]_._ ONOO^−^ can damage neurons and glial cells by lipid peroxidation and cell membrane destabilization; it can also cause DNA disintegration and subsequent poly (ADP-ribose) polymerase (PARP) activation, which leads to cell energy reduction and cell death [2]. In pneumococcal meningitis, adjuvant therapy with an ONOO^−^ scavenger reduces the number of CSF leukocytes concentrations and reduces the brain concentrations of IL-1*β* and MIP-2 [46]. This reduction is associated with a decrease of the number of leukocytes in the CSF, suggesting the involvement of ROS/RNS and proinflammatory cytokines and chemokines in the attraction of leukocytes from the blood into the subarachnoid space [3]. ONOO^−^ can contribute to the development of meningeal inflammation and increase the production of IL-8. This chemokine is equivalent to rat MIP-2; it is a chemoattractant and is involved in the migrations of leukocytes in pneumococcal meningitis [47]. In addition, treatment with a monoclonal antibody that binds with IL-8 attenuates pleocytosis in experimental pneumococcal meningitis in rabbits [48].

*In vitro*, the production of cytokines by human mononuclear cells was regulated by ONOO^−^. This activation was mediated via the transcription factor NF-*κ*B by a mechanism that may involve nitration or dephosphorylation of I*κ*B-a which leads to NF-*κ*B translocation and release of TNF-*α* [49].

One of the major and first pathologies during pneumococcal meningitis is the breakdown of the BBB. In an animal model, the BBB breakdown occurred at 12 hours after pneumococcal meningitis induction [50], subsequent to the cytokine production [35]. ROS and RNS have been implicated as mediators of the BBB breakdown [3], suggesting that the increase of the BBB permeability appears to be related to the presence of NO^•^ and O_2_^−•^ [51]. Furthermore, treatment with antioxidant prevented BBB disruption [41, 46].

Neurological sequelae from pneumococcal meningitis are estimated to occur in 30 to 52% of surviving patients [1, 52]. This damage has been demonstrated in a bacterial meningitis animal model; in this model, the surviving animals showed memory and learning impairment, depressive-like-behaviors, and anxiety-like symptoms [53]. In addition, coadjuvant treatment with antioxidants prevented cognitive impairment and oxidative stress in the brain of the survivor rats of the bacterial meningitis animal model [54]. ROS and RNS are related to these cognitive sequelae because of the cellular damage that they cause. The nervous system is a unique network of diverse cell types, comprising multiple proteins, lipids, and carbohydrates, and has important interactions with all major organs in the body [55]. Thus, the brain becomes particularly vulnerable to oxidative damage due to its high oxygen consumption, the abundance of iron, relatively low expression of antioxidants levels [55], and high presence of the polyunsaturated fatty acids [3]. H_2_O_2_ and pneumolysin produced by pneumococcus can cause neuronal cell death through mitochondrial damage [45, 56], leading to the release of apoptosis-inducing factor (AIF) into the cytosol and subsequently inducing apoptosis by a caspase-independent pathway [56]. Furthermore, leukocytes activate the tumor suppressor protein (p53) and the ataxia telangiectasia-mutated (ATM) kinase, which induce mitochondria to release cytochrome-c. Cytochrome-c, Apaf-1, and dATP/ATP are needed to form the apoptosome which is a special protein complex. Subsequently, apoptosome activates the caspase-9, that results in the activation of caspase-3 and apoptosis [56, 57]. The formation of ROS can cause direct damage through lipid peroxidation and carbonylation. Lipid peroxidation can be increased in serum [58] and in the CSF of children with bacterial meningitis [59].

## 6. Conclusion

Understanding the interactions between the complex immune network, composed of cytokines, chemokine, leukocytes, and oxidative stress, and bacterial virulence factors may help to establish more effective therapeutic strategies for CNS infections and, therefore, a better outcome for affected subjects.

## Abbreviations

AIF: Apoptosis-inducing factor
AIM2: Absent in melanoma 2
Apaf: Apoptosis protease activating factor
ASC: apoptosis speck-like protein
ATM: Ataxia telangiectasiamutated
BBB: Blood-brain barrier
CNS: Central nervous system
CSF: Cerebrospinal fluid
CXCL-8: Interleukin 8
DNA: Desoxyribonucleic acid
Fe^2^: Iron ion
H_2_O_2_: Hydrogen peroxide
ICAM: Intercellular adhesion molecule
IkB: Immunome knowledge base
IKK: Immunome knowledge base kinase
IL: Interleukin
IL-1Rs: interleukin receptors
iNOS: Inducible nitric oxide synthase
IRAK4: IL-1 receptor-associated kinase-4
LFA: Lymphocyte-function-associated antigen
MAPKKK: Mitogen-activated protein kinase kinase kinase
MIP-2: Macrophage inflammatory protein
MyD88: Myeloid differentiation factor 88
NADPH: Nicotinamide adenine dinucleotide phosphate
NF-*κ*B: Nuclear factor kappa B
NLR: NOD-Like receptor
NO: Nitric oxide
NOD/CARD: Nucleotide oligomerization domain/caspase recruitment domain
O_2_^−•^: Superoxide anion radicals
OCl: Hypochlorite
OH: Hydroxyl radicals
ONOO*‒*: Peroxynitrite
PAF: Platelet-activating-factor
PAMP: Pathogen-associated molecular patterns
PARP: Poly-ADP-ribose polymerase
pIgR: Polymeric Ig receptor
PRRs: Pattern recognition receptors
PspC: Protein C pneumococcal surface
RNS: Reactive nitrogen species
ROS: Reactive oxygen species
*S. pneumoniae*: *Streptococcus pneumoniae*
SOD: Superoxide dismutase
TAK1: TGF-*β*-activated kinase 1
TLR: Toll-like receptor
TRAF6: Tumor necrosis factor receptor associated factor 6.
